# Powered Respirators Are Effective, Sustainable, and Cost-Effective Personal Protective Equipment for SARS-CoV-2

**DOI:** 10.3389/fmedt.2021.729658

**Published:** 2021-10-14

**Authors:** Alasdair Munro, Jacqui Prieto, Emmanouil Mentzakis, Mohammed Dibas, Nitin Mahobia, Peter Baker, Sarah Herbert, Trevor Smith, Matthew Hine, Joann Hall, Angie McClarren, Mike Davidson, Julie Brooks, Jane Fisher, David Griffiths, Hywel Morgan, Corrado Giulietti, Saul N. Faust, Paul Elkington

**Affiliations:** ^1^NIHR Southampton Clinical Research Facility and NIHR Southampton Biomedical Research Centre, University Hospital Southampton NHS Foundation Trust, Southampton, United Kingdom; ^2^Faculty of Medicine and Institute for Life Sciences, University of Southampton, Southampton, United Kingdom; ^3^School of Health Sciences and Institute for Life Sciences, University of Southampton, Southampton, United Kingdom; ^4^PeRSo Implementation Team, University Hospital Southampton NHS Foundation Trust, Southampton, United Kingdom; ^5^School of Economics, Social and Political Science, University of Southampton, Southampton, United Kingdom; ^6^Faculty of Engineering & Physical Sciences and Institute for Life Sciences, University of Southampton, Southampton, United Kingdom

**Keywords:** COVID-19, respirator, healthcare, personal protective equipment, economic analysis, nosocomial infection

## Abstract

**Objectives:** The provision of high-quality personal protective equipment (PPE) has been a critical challenge during the COVID-19 pandemic. We evaluated an alternative strategy, mass deployment of a powered air-purifying respirator (PeRSo), in a large university hospital.

**Methods:** We performed prospective user feedback *via* questionnaires sent to healthcare workers (HCWs) issued PeRSos, economic analysis, and evaluated the real-world impact.

**Results:** Where paired responses were available, PeRSo was preferred over droplet precautions for comfort, patient response, overall experience, and subjective feeling of safety. For all responses, more participants reported the overall experience being rated “Very good” more frequently for PeRSo. The primary limitation identified was impairment of hearing. Economic simulation exercises revealed that the adoption of PeRSo within ICU is associated with net cost savings in the majority of scenarios and savings increased progressively with greater ITU occupancy. In evaluation during the second UK wave, over 3,600 respirators were deployed, all requested by staff, which were associated with a low staff absence relative to most comparator hospitals.

**Conclusions:** Health services should consider a widespread implementation of powered reusable respirators as a safe and sustainable solution for the protection of HCWs as SARS-CoV-2 becomes an endemic viral illness.

## Introduction

In early 2020, the emerging respiratory virus epidemic, which was first identified in China, rapidly spread across the world (1). While there was some initial uncertainty about the mode of transmission of SARS-CoV-2, a consensus has emerged that airborne transmission plays an important role (2). In countries affected early in the pandemic, high rates of infection among healthcare workers (HCWs) were reported, with notable deaths of relatively young members of staff, despite an otherwise strong age-dependent effect on mortality (3). The urgent demand for personal protective equipment (PPE) quickly overwhelmed healthcare services, leading to severely stretched supply chains and rationing of supplies (4).

More recently, new variants of the SARS-CoV-2 virus have emerged, which show signs of antigen escape (5), leading to projections that the virus may become an endemic, seasonal disease (6). Consequently, there is an emerging need for long-term, sustainable PPE solutions with high efficacy to protect HCW from infection.

Powered air-purifying respirators (PAPRs) are an alternative PPE approach to standard disposable face masks and are recognized to provide a higher degree of protection than FFP3 (or equivalent N95) facemasks by regulatory bodies such as the UK Health and Safety Executive (HSE) and US Center for Diseases Control (7, 8). In response to the urgent need to protect HCW and find safe, sustainable solutions to the PPE supply chain crisis, a collaboration was formed between respiratory physicians at University Hospital Southampton (UHS), the Engineering department at the University of Southampton (UoS), and a local electronics company (INDO, part of the Baynhams group). A design for a personal respirator, manufactured from cheap and readily available materials, was developed and prototypes produced, with the design made available open source (9). They comprise a battery-operated fan held on a belt, which draws air in through a high-efficiency particulate air (HEPA) filter and delivers clean air *via* a corrugated tube into an overhead hood with a clear, plastic visor (subsequent editions of the hood had an “over ear” fit). They can be worn for extended periods of time, and battery life is up to 8 h and batteries can be changed while on shift. Within 4 weeks, mass production commenced by modifying a commercially available industrial respirator for healthcare use, and the Personal Respirator Southampton (PeRSo) was deployed for routine use within our large NHS hospital. The respirators in use have been given full certification for use as alternative to FFP3 masks, achieving approval against BS EN1291, and also conform to EU 2016/425. During the first UK peak between April and May 2020, 1,896 respirators were issued, and during the second peak in January–February 2021, 3,632 were deployed. The PeRSos were issued on an individual basis to staff members, which were then available for their personal use at work 24 h a day, 7 days a week.

The implementation process was complex, once the strategy was approved by the hospital executive group, requiring a large project group to address all logistical aspects. Considerations included procurement, manufacture, design modification, delivery, power supply (for up to 5,000 units charging on-site during pandemic emergency periods), storage during deployment and after use, infection control and cleaning, education, communication, and evaluation. To assess the implementation and suitability for widespread use in healthcare settings, we gathered early feedback on the deployment of the PeRSo by the end users (all types of hospital staff) to inform future designs and processes. As protection from SARS-CoV-2 is likely to be a long-term requirement, we also evaluated the economic impacts of PeRSo use relative to standard NHS PPE solutions. Finally, we analyzed the real-world impact during the widespread use in the second wave.

## Methods

**Figure 1:**
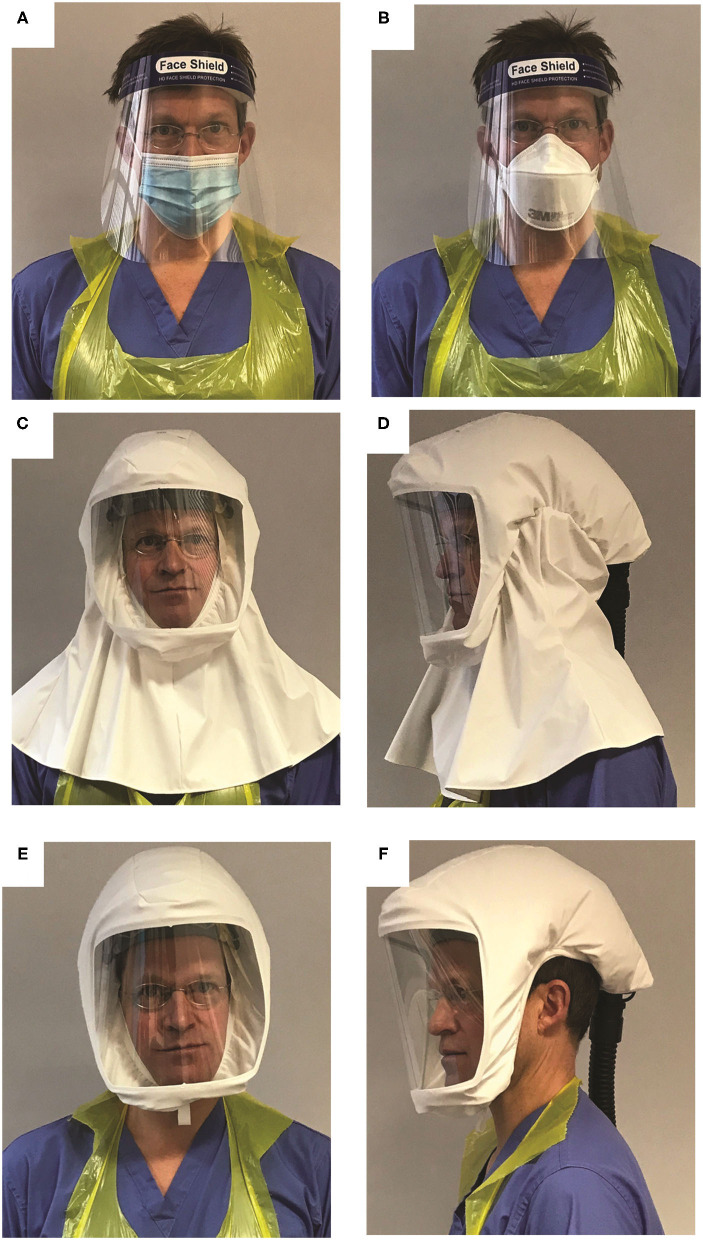
Types of PPE described in the manuscript. **(A)** Droplet protection: a disposable Type IIR surgical mask and visor. **(B)** Aerosol protection: a disposable tight fitting FFP3 mask and visor. **(C,D)** PeRSo with the long hood type, which was the type issued during the period of the survey. **(E,F)** PeRSo with short hood type that exposes the ears, developed in response to the initial user feedback. In all instances, a plastic apron and gloves complete the PPE.

While awaiting HSE approval for use as a replacement against an FFP3 mask (requiring BS EN12941), PeRSos were issued in a pilot deployment in replacement of droplet precaution PPE (surgical facemask plus eye protection/visor, Figure 1). This PPE was the standard on wards with COVID-confirmed patients when no aerosol-generating procedures were being undertaken, and so PeRSos were first deployed on the “red” COVID wards. Each PeRSo was allocated to a staff member at UHS based on the risk of exposure and current supplies of PPE, so allocations were initially to staff from wards caring for confirmed SARS-CoV-2-positive patients, followed by emergency department staff caring for patients on unknown infection status. The roll-out for wave 1 was from April 21, 2021, to August 6, 2021, issuing a total of 1,896 respirators that were then recalled for checking and storage, and the second roll-out was from November 2, 2020–present, to date issuing a total of 3,632 respirators, all individually requested by staff.

### Rapid Feedback Survey

Each user received dedicated training on the operation, care, and “donning” and “doffing” (putting on and removing) the PeRSo and provided details so they could be contacted with an e-survey. The first survey was issued within 24 h, regarding feedback on the use of standard issue PPE (Figure 1A). If the participant spent more than, or equal to, 10 h per week in airborne precaution PPE (including FFP3 respirator, full facial visor, fluid-resistant gown, and gloves, Figure 1B), then they were asked specifically about their experiences of airborne PPE. Otherwise, participants were asked regarding their experiences of using droplet precaution PPE (surgical mask, eye protection/visor, plastic apron, and gloves). The surveys included questions on comfort, ease of use, impact on communication, and HCW impressions of the patient experience. These were answered on an ordinal scale with five possible answers. If participants reported a negative experience, they were prompted to provide a free text response to explain why. Additional free text space was available at the end of the survey for miscellaneous comments. After 72 h, participants were sent a second e-survey regarding their feedback on the use of the PeRSo (Figures 1C,D). The same questions were issued about all types of PPE for comparison.

Survey results were collated and presented as tallies with percentages. Where paired responses were available (the same respondent replying to both surveys), a statistical analysis was performed separately using a Wilcoxon signed rank test. Responses were converted to an ordinal scale of 1–5 (1 being worst, 5 being best) for the analysis. Free text comments were downloaded from the online questionnaire individually and classified by an investigator into a series of groups based on the themes that emerged, including classification as either “positive” or “negative” comments, presented with a qualitative summary. The qualitative data analysis was performed to support and enhance the quantitative analysis, and a second investigator reviewed and cross-checked the classification by the initial scorer.

### Patient Feedback

Patients were surveyed on their preferences by an informal survey, due to infection prevention constraints in place at the peak of the first wave limiting additional contact with ward-based patients. A laminated card with pictures of HCW wearing either airborne PPE or the PeRSo (Supplementary Appendix—Figure A) was presented, and the patients were asked to point to which they would prefer, or if they had no preference, and any general comments were also collated. Results were tallied and presented as descriptive statistics only.

### Economic Analysis: Comparing PeRSo and PPE Costs

A simulation exercise was performed to understand the financial impact of full adoption of PeRSo in place of standard PPE within a 20-bedded intensive care unit. The simulation provides a cost comparison across a range of scenarios over a time horizon of 360 days and with the number of intensive care patients varying from 5 to 40. Separate calculations are presented for two possible prices of PeRSo. The model includes the life span of the equipment (hoods and filters requiring replacement every 6 months). The outcome of the analysis was the cumulative daily PeRSo cost saving (pounds sterling), corresponding to the difference between the PPE costs and the PeRSo costs. PeRSo cost saving is positive (negative) when PPE costs are greater (smaller) than PeRSo costs. A full description of the scenarios and a detailed explanation of the assumptions within the model are included in the Supplementary Appendix.

### Analysis of Impact During the Second UK Wave

Staff requests for PeRSos and total issue numbers were collected prospectively. Staff absence rates due to COVID were analyzed from nationally available datasets and compared with equivalent large NHS teaching hospitals across England. The average daily staff absence due to COVID-19 was calculated per 1,000 clinically active staff for each hospital.

### Ethics

The user and patient feedback surveys were conducted as a service evaluation exercise; therefore, formal ethical approval was not required as per local legislation. The economic and staff absence analysis was performed using publicly available data so no formal ethical approval was required.

## Results

### Survey Responses

A total of 760 invites were sent for each survey, of which 140 completed the survey on standard issue PPE and 170 completed the survey regarding PeRSo. Most respondents to each survey were female. The most common age for each survey was 25–30, and the most common role was nursing (Table 1). Results of the survey responses are presented in Tables 2, 3.

**Table 1:** Demographics of respondents.

	**Standard PPE**	**PeRSo**
**Gender**		
Female	101/140 (72.1%)	133/172 (77.3%)
**Age**		
<25 y	17/140 (12.1%)	14/172 (8.1%)
25–30 y	37/140 (26.4%)	43/172 (24.9%)
30–35 y	30/140 (21.4%)	37/172 (21.4%)
35–40 y	16/140 (11.4%)	25/172 (14.5%)
40–50 y	31/140 (22.1%)	38/172 (22%)
>50 y	9/140 (6.4%)	16/172 (9.3%)
**Role**		
Allied health professional	14/140 (10%)	14/172 (8.1%)
Advanced nurse practitioner	4/140 (2.9%)	5/172 (2.9%)
Healthcare assistant	23/140 (16.4%)	36/172 (20.9%)
Nurse	48/140 (34.3%)	59/172 (34.3%)
Doctor	34/140 (24.3%)	39/172 (22.7%)
Other	17/140 (12.1%)	19/172 (11%)

**Table 2:** Survey responses.

	***N***	**Very uncomfortable**	**Quite uncomfortable**	**Neutral**	**Quite comfortable**	**Very comfortable**
**How comfortable is the PPE to wear?**						
Droplet	82	2 (2.4%)	27 (32.9%)	30 (36.6%)	18 (22%)	5 (6.1%)
Airborne	45	3 (6.7%)	12 (26.7%)	13 (28.9%)	15 (33.3%)	2 (4.4%)
PeRSo	147	4 (2.7%)	25 (17%)	33 (22.5%)	74 (50.4%)	11 (7.5%)
**How easy is it to don (put on) the PPE?**						
	***N***	**Very difficult**	**Quite difficult**	**Neutral**	**Quite easy**	**Very easy**
Droplet	82	0 (0%)	4 (4.9%)	11 (13.4%)	41 (50%)	26 (31.7%)
Airborne	45	0 (0%)	1 (2.2%)	15 (33.3%)	25 (55.6%)	4 (8.9%)
PeRSo	146	0 (0%)	5 (3.4%)	29 (19.9%)	86 (58.9%)	26 (17.8%)
**How easy is it to doff (take off) the PPE?**						
	***N***	**Very difficult**	**Quite difficult**	**Neutral**	**Quite easy**	**Very easy**
Droplet	81	0 (0%)	6 (7.4%)	11 (13.6%)	42 (51.9%)	22 (27.2%)
Airborne	45	0 (0%)	9 (20%)	9 (20%)	22 (48.9%)	5 (11.1%)
PeRSo	146	0 (0%)	17 (11.6%)	38 (26%)	82 (56.2%)	9 (6.2%)
**How would you describe patient's responses to the PPE?**						
	***N***	**Responded very badly**	**Responded quite badly**	**Neutral**	**Responded quite well**	**Responded very well**
Droplet	82	0 (0%)	4 (4.9%)	43 (52.4%)	31 (37.8%)	4 (4.9%)
Airborne	45	0 (0%)	3 (6.8%)	21 (47.7%)	17 (38.6%)	3 (6.8%)
PeRSo	147	0 (0%)	4 (2.7%)	56 (38.1%)	80 (54.4%)	7 (4.7%)
**Which statement best describes your subjective feeling of safety while using the PPE?**						
	***N***	**I feel very unsafe**	**I feel quite unsafe**	**Neutral**	**I feel quite safe**	**I feel very safe**
Droplet	82	4 (4.9%)	21 (25.6%)	21 (25.6%)	28 (34.2%)	8 (9.8%)
Airborne	45	1 (2.2%)	8 (17.8%)	7 (15.6%)	25 (55.6%)	4 (8.9%)
PeRSo	147	0.0%	4 (2.7%)	22 (15%)	71 (48.3%)	50 (34%)
**How would you rate the overall experience of wearing the PPE?**						
	***N***	**Very poor**	**Quite poor**	**Neutral**	**Quite good**	**Very good**
Droplet	82	3 (3.7%)	12 (14.6%)	40 (48.8%)	23 (28.1%)	4 (4.9%)
Airborne	45	2 (4.4%)	8 (17.8%)	12 (26.7%)	20 (44.4%)	3 (6.7%)
PeRSo	147	2 (1.4%)	14 (9.5%)	36 (24.5%)	74 (50.3%)	21 (14.3%)
**Which statement best describes your experiences of speaking to others while wearing the PPE?**						
	***N***	**Have to shout**	**Have to raise voice significantly**	**Have to raise voice moderately**	**Have to raise voice mildly**	**Speak normally**
Droplet	77	3 (3.9%)	14 (18.2%)	32 (41.6%)	25 (32.5%)	3 (3.9%)
Airborne	39	1 (2.6%)	12 (30.8%)	19 (48.7%)	7 (18%)	0 (0%)
PeRSo	143	4 (2.8%)	40 (27.8%)	56 (39.2%)	36 (25.2%)	7 (4.9)%
**Which statement best describes your vision while wearing the PPE** ***?***						
	***N***	**Extremely impaired**	**Very impaired**	**Somewhat impaired**	**Mildly impaired**	**Normal vision**
Droplet	77	0 (0%)	7 (9.1%)	18 (23.4%)	20 (26%)	32 (41.6%)
Airborne	39	0 (0%)	3 (7.7%)	7 (18%)	18 (46.2%)	11 (28.2%)
PeRSo	144	1 (0.7%)	5 (3.5%)	26 (18.1%)	48 (33.3%)	64 (44.4%)
**Which statement best describes your experience of hearing while wearing the PPE?**						
	***N***	**Extremely impaired**	**Very impaired**	**Somewhat impaired**	**Mildly impaired**	**Normal hearing**
Droplet	77	3 (3.9%)	7 (9.1%)	25 (32.5%)	13 (16.9%)	29 (37.7%)
Airborne	39	2 (5.1%)	11 (28.2%)	13 (33.3%)	8 (20.5%)	5 (12.8%)
PeRSo	143	12 (8.4%)	39 (27.3%)	70 (49%)	19 (13.3%)	3 (2.1%)

**Table 3:** Statistical analysis of paired survey responses (Wilcoxon signed rank).

	***N***	**Very uncomfortable**	**Quite uncomfortable**	**Neutral**	**Quite comfortable**	**Very comfortable**	**Median difference (95% CI)**	***P***
**How comfortable is the PPE to wear?**								
Droplet	35	0 (0%)	12 (34.3%)	13 (37.1%)	9 (25.7%)	1 (2.9%)	−1 (−1.5, −0.4)	0.011^*^
PeRSo		1 (2.9%)	4 (11.4%)	8 (22.9%)	18 (51.4%)	4 (11.4%)		
Airborne	14	1 (7.1%)	4 (28.6%)	4 (28.6%)	5 (35.7%)	0 (0%)	−1.5 (−2.5, 0.5)	0.087
PeRSo		0 (0%)	2 (14.3%)	1 (7.1%)	11 (78.6%)	0 (0%)		
**How easy is it to don (put on) the PPE?**								
	***N***	**Very difficult**	**Quite difficult**	**Neutral**	**Quite easy**	**Very easy**	**Median difference (95% CI)**	***p***
Droplet	35	0 (0%)	0 (0%)	3 (8.6%)	19 (54.3%)	13 (37.1%)	1 (0, 1.5)	0.06
PeRSo		0 (0%)	4 (11.4%)	2 (5.7%)	21 (60%)	8 (22.9%)		
Airborne	14	0 (0%)	0 (0%)	6 (42.9%)	7 (50%)	1 (7.1%)	−1 (-1.5, 2.4)	0.095
PeRSo		0 (0%)	0 (0%)	1 (7.1%)	11 (78.6%)	2 (14.3%)		
**How easy is it to doff (take off) the PPE?**								
	***N***	**Very difficult**	**Quite difficult**	**Neutral**	**Quite easy**	**Very easy**	**Median difference (95% CI)**	***p***
Droplet	35	0 (0%)	0 (0%)	3 (8.6%)	21 (60%)	11 (31.4%)	1.5 (1, 2)	<0.001^*^
PeRSo		0 (0%)	6 (17.1%)	4 (11.4%)	24 (68.6%)	1 (2.9%)		
Airborne	14	0 (0%)	3 (21.4%)	4 (28.6%)	6 (42.9%)	1 (7.1%)	−2 (1.5, 1)	0.916
PeRSo		0 (0%)	2 (14.3%)	5 (35.7%)	7 (50%)	0 (0%)		
**How would you describe patient's responses to the PPE?**								
	***N***	**Responded very badly**	**Responded quite badly**	**Neutral**	**Responded quite well**	**Responded very well**	**Median difference (95% CI)**	***p***
Droplet	35	0 (0%)	2 (5.7%)	20 (57.1%)	13 (37.1%)	0 (0%)	−1 (−1, 0)	0.044^*^
PeRSo		0 (0%)	2 (5.7%)	11 (31.4%)	19 (54.3%)	3 (8.6%)		
Airborne	14	0 (0%)	1 (7.1%)	7 (50%)	6 (42.9%)	0 (0%)	−1 (−1, 0)^**^	0.182
PeRSo		0 (0%)	0 (0%)	6 (42.9%)	7 (50%)	1 (7.1%)		
**Which statement best describes your subjective feeling of safety while using the PPE?**								
	***N***	**I feel very unsafe**	**I feel quite unsafe**	**Neutral**	**I feel quite safe**	**I feel very safe**	**Median difference (95% CI)**	***p***
Droplet	35	2 (5.7%)	13 (37.1%)	10 (28.6%)	10 (28.6%)	0 (0%)	−2 (−2.5, −1.5)	<0.0001^*^
PeRSo		0 (0%)	1 (2.9%)	3 (8.6%)	14 (40%)	17 (48.6%)		
Airborne	14	0 (0%)	5 (35.7%)	2 (14.3%)	7 (50%)	0 (0%)	−1.5 (−2, −1)	0.007^*^
PeRSo		0 (0%)	0 (0%)	2 (14.3%)	8 (57.1%)	4 (28.6%)		
**How would you rate the overall experience of wearing the PPE?**								
	***N***	**Very poor**	**Quite poor**	**Neutral**	**Quite good**	**Very good**	**Median difference (95% CI)**	***p***
Droplet	35	1 (2.9%)	7 (20%)	18 (51.4%)	9 (25.7%)	0 (0%)	−1 (−1.5, −0.5)	0.006^*^
PeRSo		0 (0%)	5 (14.3%)	11 (31.4%)	12 (34.3%)	7 (20%)		
Airborne	14	0 (0%)	4 (28.6%)	6 (42.9%)	4 (28.6%)	0 (0%)	−1 (−1.5, 0.5)	0.168
PeRSo		1 (7.1%)	1 (7.1%)	2 (14.3%)	10 (71.4)	0 (0%)		

*A negative median difference indicates a response in favor of the PeRSo*.

* *indicates a statistically significant difference*.

** *should have legend: 60% Confidence interval due to sample size and variability*.

Statistically significant findings were that more participants reported that PeRSo use was “Quite comfortable” (50.4%), as compared to droplet (22%) or airborne (33.3%) precautions. The PeRSo was reported to be “Very easy” to don more frequently than airborne precautions (17.8 vs. 8.9%), but less frequently than droplet (31.7%). Doffing was reported to be very easy less frequently for the PeRSo (6.2%) than either airborne (11.1%) or droplet precautions (27.2%). Patients were reported to have responded “Quite well” to the PeRSo (54.5%) more often than airborne (38.6%) or of droplet precautions (34.2%), although a similar number reported patients responded “Very well” for each. The overall experience of wearing the PeRSo was reported as “Very good” (14.3%) more often than either airborne (6.7%) or droplet precautions (4.9%).

More participants reported having to “Raise their voice significantly” for both PeRSo (27.8%) and airborne (30.8%) precautions compared to droplet (18.2%). The vision was reported as “Normal” most frequently for PeRSo (44.4%) and droplet (41.6%) compared to airborne precautions (28.2%). The hearing was reported as “Very impaired” more frequently for PeRSo (27.3%) and airborne precautions (28.2%) than for droplet precautions (9.1%).

Paired responses were available for 35 participants to compare responses for PeRSo and droplet precautions, and 14 to compare PeRSo and airborne precautions, permitting comparative analysis using the Wilcoxon signed rank test (Table 3). PeRSo was significantly favored compared to droplet precautions for comfort (*p* = 0.011), patient responses (*p* = 0.044), subjective feeling of safety (*p* = < 0.001), and overall experience (*p* = 0.006). Compared to airborne precautions, the small number of participants familiar with this PPE limited the power, but again a greater subjective feeling of safety was reported for PeRSo (*p* = 0.007).

### Free Text Comments

Analysis of the free text feedback of the questionnaire was performed to give contextual information to support the quantitative analysis and identify recurrent themes. In the positives relating to PeRSo use, these primarily centered on the greater comfort and sense of security. For droplet masks, the majority of comments were negative (16: e.g., “hurts my ears,” “uncomfortable”), and similarly, for airborne masks, most were negative (13: e.g., “suffocating,” “claustrophobic”), whereas for PeRSo most were positive (12: “comfortable,” “nice cool air”). No positive responses for comfort were reported for standard PPE. For the perception of safety, again comments were consistent with the quantitative data, with comments such as “I don't feel safe” and “I doubt it is effective” for droplet PPE, “feel unsafe” and “inadequate” for airborne PPE, vs. a predominant free text entry of “feels very safe” or similar in 25 responses for PeRSo.

The negatives of PeRSo use mainly focused on the noise and communication difficulties related to the full-length hood that covered the ears (Figures 1C,D), with issues related to noise reported by 21 respondents. Other free text comments were balanced between positives “the patient can see my smile,” “the visor fogs less,” and negatives, such as “nowhere to store on wards” and “loss of peripheral vision.”

### Patient Feedback

Inpatients on the general respiratory and elderly care wards were approached as part of the service evaluation and shown an image of three staff members in either standard airborne PPE or PeRSo hoods (Supplementary Figure A). They were then asked which type of PPE they preferred, or if no preference, and any general comments. The outcome was 32 selected PeRSo, 20 chose standard PPE, with eight no preference. Of those who chose standard PPE, the main reason reported was uncertainty whether the alternative PPE would be effective, whereas being able to see the face and the consequent improvement in communication and showing of empathy was the main reason for selecting PeRSo.

### Economic Analysis

**Figure 2:**
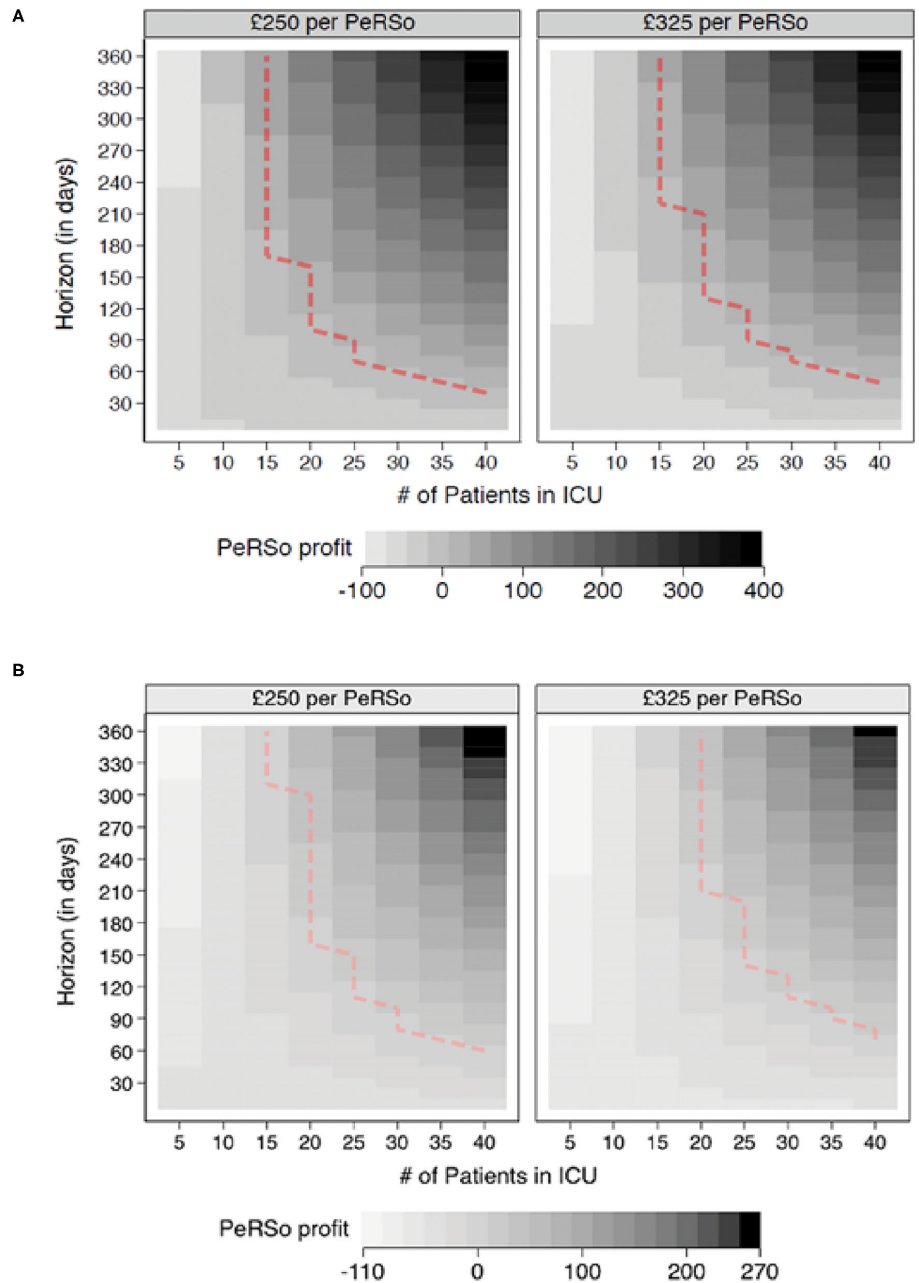
PeRSo cost savings. **(A)** Comparisons between PeRSo and FFP3 facemasks. **(B)** Comparisons between PeRSo and a mixture of FFP3 and surgical facemasks. Positive values represent simulation outcomes where PPE costs are greater than PeRSo costs. The dashed line represents the boundary of PeRSo deployment becoming cost saving, with outcomes further to the right implying greater saving from PeRSo use.

**Figure 3:**
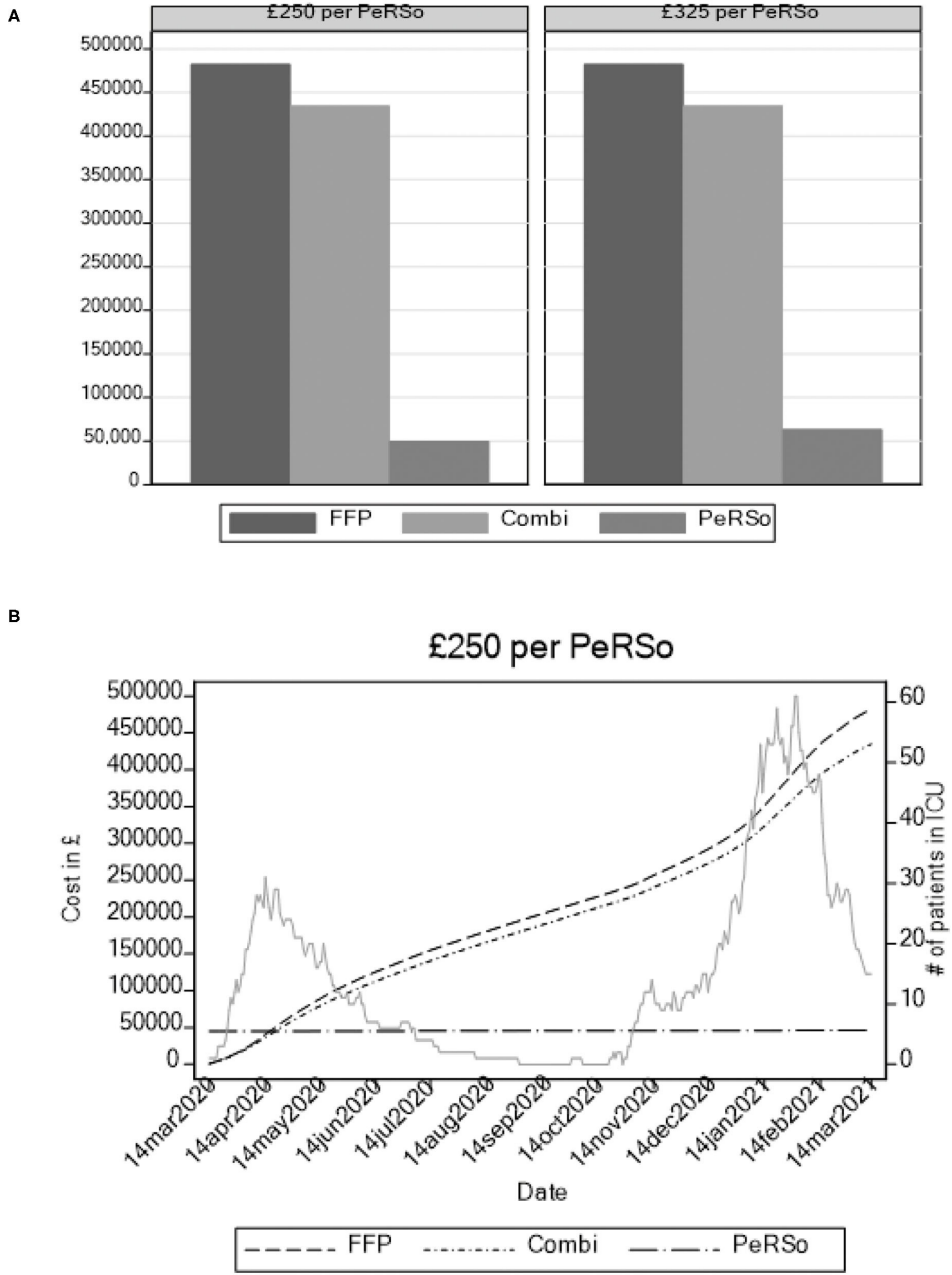
Modeling of cumulative annual cost of three PPE strategies based on admission rates at University Hospital Southampton intensive care unit admissions. **(A)** Total cost accumulated over 12 months. **(B)** Cumulative costs over time, with ITU admission rate plotted in background to demonstrate waves.

To determine whether mass PeRSo use would be cost-effective, a simulation exercise was performed. This demonstrated that over the time horizon of 360 days, with scenarios modeling a constant ICU patient population fixed at values from 5 to 40, the adoption of PeRSo is associated with net cost savings in the majority (57.8%) of scenarios, rising to 100% when considering scenarios involving 15 or more ICU patients. The central finding was that PeRSo net cost savings increase with the number of patients on intensive care and with the length of time from deployment. Heat maps summarize the key patterns of PeRSo net savings according to time and bed occupancy (Figure 2). The vertical axis of the graphs represents the time horizon in days, and the horizontal axis the number of patients. Darker cells represent greater PeRSo cost saving, with the dashed line representing the boundary of PeRSo net cost vs. saving. PeRSo use becomes cost saving more rapidly in the scenario where PPE consists of 100% FFP3 respirators, achieving cost neutrality at 90 days when 20 beds are occupied, and becoming progressively more cost savings thereafter (Figure 2A). When PPE consists of a mixture of FFP3 respirators and surgical facemasks, cost neutrality for PeRSo use is reached at 155 days, when 20 beds are occupied (Figure 2B). In both panels, two alternative costs for PeRSo (£250 and £325) are considered, to demonstrate the impact of greater initial investment cost. The NHS for bulk purchase is likely to be at the lower range, but for completeness, we performed an analysis at two price points within the likely unit price range. To validate this model with the real-world data, we then performed a retrospective analysis based on our experience at UHS (Figures 3A,B). Initial costs were high, as almost all costs occur at the initial deployment, but then savings progressively accumulated as the pandemic progressed, as ongoing costs are much less than disposable PPE, with cost saving increasing progressively during wave 2 of the pandemic (Figure 3B).

### Widespread Respirator Use Associates With Low Staff Absence Rates

**Figure 4:**
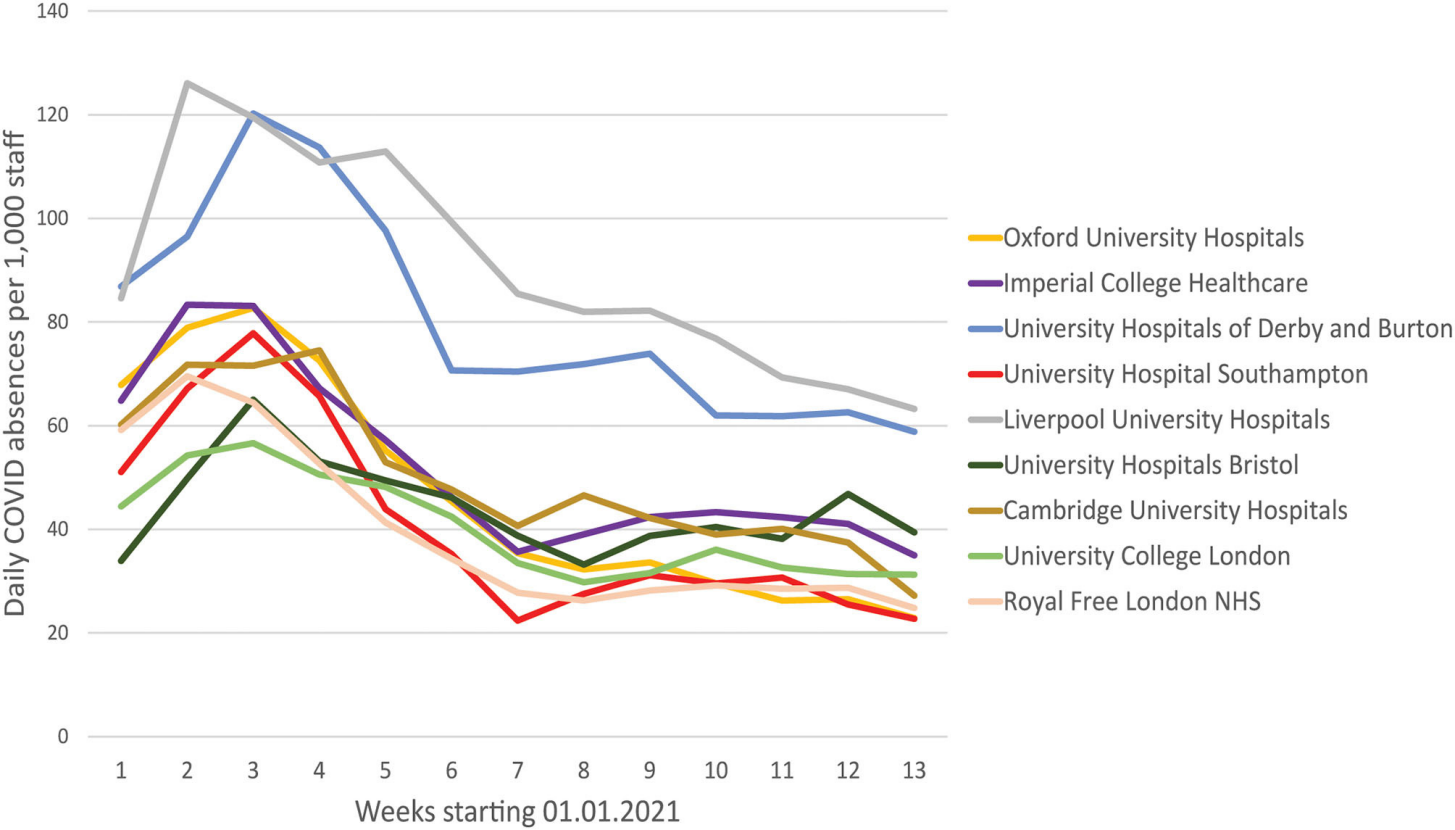
Staff absence rates due to COVID19 were lower in Southampton than equivalent large UK teaching hospitals. Average daily absence rates per 1,000 clinically active staff are presented for the first quarter of 2021, as reported by individual hospital trusts to the national database. University Hospital Southampton is red, and the initial peak in the New Year rapidly fell, and remained low, coinciding with the progressive roll-out of PeRSo respirators.

Finally, the second UK wave of the pandemic from November 2020 permitted the real-world evaluation of the impact of mass deployment. The protocol for the PeRSo issue was changed, whereby staff were asked to request the issue of a respirator by e-mailing a central distribution hub, in contrast to the ward-based deployment used for the first wave. Over 3,600 staff requested a respirator, out of a total of 6,431 clinically active staff across the hospital. Therefore, the initial positive feedback reported in wave 1 was maintained as high-level staff requests for respirators in wave 2, as many of the staff not requesting PeRSos would have been working in areas where respirator use was not recommended. The distribution center needed to operate 7 days per week during late December and early January to meet demand. To determine whether respirator use may increase or reduce staff infection, we analyzed staff absence due to COVID-19 in hospitals in England with between 6,000 and 8,000 clinically active staff. We demonstrated that overall infection rates were low compared to most comparator large NHS teaching hospitals (Figure 4). Any absence related to COVID-19, including the requirement to self-isolate due to a family member being infected, is recorded in this NHS database. In the New Year, there was a sharp peak in absences in most NHS hospitals, but this rapidly fell in Southampton Hospital over the same period that PeRSo use increased (Red line), with a 3.4-fold drop in absences over 4 weeks after the peak compared to an average 1.9-fold fall in the comparator hospitals. The worst-performing hospital had 2.8 times the staff absence over the pandemic period relative to University Hospital Southampton. Therefore, PeRSo use maximized staff availability, as in addition the 5% of staff who fail FFP3 fit testing were able to return to work.

## Discussion

The COVID-19 pandemic resulted in an unprecedented demand for PPE to protect HCW around the world (10), as hospitals rapidly filled with acutely unwell patients suffering from a new respiratory virus with airborne transmission (2). Health services were quickly overwhelmed by demand and PPE procurement became a matter of international importance (11) and a matter of much contention (12). The demonstration that coughing is a major source of aerosol generation (13) has led to calls for the much wider provision of respiratory protective equipment (RPE) for HCW than current national recommendations (14). Alongside traditional supply chains, more sustainable solutions are required to provide HCW with the highest possible degree of protection and to ensure that demands for PPE do not outstrip supply, resulting in rationing and avoidable HCW infection (15).

We successfully launched a pilot deployment of new PAPR equipment in a large university hospital during the first wave of the United Kingdom epidemic to replace droplet PPE, which does not protect against aerosol transmission (16). This presented a logistical challenge, and the project management involving stakeholders from health care, academia, and industry, necessitating cross-sector co-ordination (Box 1). The short timeframe within which the design and manufacturing process was undertaken demonstrates the potential of widespread PAPR use within a pandemic as an alternative PPE strategy. While our institution opted for deployment at an individual staff member level (i.e., one allocated personally to a staff member), we are aware of other institutions who have successfully utilized a PAPR “library,” for cycled allocation with cleaning between use among staff members. One option that minimizes costs without increasing the risk of cross-contamination is to supply each staff member a hood with a breather tube attached and at the start of the shift issue a charged blower unit from a central repository, to which they return at the end of the shift. Reusable tight-fitting elastomeric respirators have been used as an alternative solution, which gives high levels of protection but have issues of skin pressure and communication challenges due to the mouth not being visible and muffling effect, and so there is a trade-off between compactness of reusable device vs. comfort and communication.

Box 1 **Stakeholders and implementation tasks.**

**Table:** 

**Stakeholder**	**Role and responsibilities**
Director and nursing leads for infection prevention and control	Protocols for use, donning and doffing, assessment of usage areas
Medical and nursing director	Prioritizing staff for roll-out; ensuring compliance with regulatory guidance
Communications	Updating all staff on deployment and prioritization strategy of new PPE; news release to inform public
Logistics and estates	Deployment centers, storage areas, charging stations
Education team	Training staff in use, cleaning, storage, return at the end of contract
Procurement and purchasing	Confirming contract and delivery schedule, liaising with design team for technical aspects of manufacture, replacement hoods, spare batteries, on-site storage arrangements
Local industry	Production of units, shipping in parts, regulatory approvals
University	Initial concept and prototype evaluation; prospective analysis of deployment
End users: Doctors, nurses, healthcare assistants, research teams, phlebotomists, cleaning staff, porters	Compliance with training, storage, ongoing use, return when leaving post

Powered air-purifying respirators have several benefits over standard PPE. A PAPR such as the PeRSo is recognized as the highest standard of protection of PPE by regulators such as the UK Health and Safety Executive (7) and the US Center for Disease Control (8). First, the HEPA filter has significantly higher filtration efficacy comparted to FFP3/N95 respirators, removing over 99.9% of airborne particles (17). Second, as fit testing is not required, this allows the 5% of members of staff for whom no compatible mask is available to be part of the frontline workforce and do not need to be redeployed to other areas (18). Third, PAPRs do not fail during aerosol-generating activity, such as resuscitation (19), which frequently occurs with standard PPE due to movement and loss of the skin seal (20). Fourth, eye protection is integral to a PAPR, preventing accidental facial touching, which is likely a cause of transmission (21). Finally, PAPRs do not cause pressure sores and skin issues that FFP3 masks often cause with prolonged use (22).

Our rapid evaluation shows that the PeRSo was well-received by HCW on its initial pilot deployment, and this translated to high uptake across the hospital in COVID-facing roles during the second wave. The PeRSo was favored for its comfort and feeling of safety and was also noted to be well-received by patients. This was predominantly due to the ability to see the faces of HCW, to support patients who communicate by lip-reading, which is important in over 10% of patients, and for more elderly patients with dementia, to improve communication (23). The difficulties reported in the survey were predictable, with fan noise impairing hearing and the added inconvenience of donning and doffing being reported most frequently. We resolved the noise issue by modification the hood for the second UK wave, with a sleeker off-ear design (Figures 1E,F). Overall, despite these inconveniences reported in the first roll-out, these were significantly outweighed by the benefits felt by the wearer, as it was favored for overall experience.

As well as being preferred by both staff and patients, PeRSo deployment was also cost-effective, as our simulation exercise demonstrates. PPE has cost the UK government over £20 billion during the pandemic to date (24). The cumulative cost of PeRSo deployment is essentially driven by the fixed cost of the equipment, which is borne once and does not recur. Because of its “re-usability,” PeRSo costs are virtually independent of the number of patients and are mainly a function of staff numbers. In contrast, PPE costs are recurrent, even with sessional use, and depend both on staff numbers and the number of patients. Hence, the cost savings progressively accumulate over time and more rapidly with the number of inpatients. The wave-like nature of pandemics creates challenges to accurate modeling, with the need and costs of PPE changing over time, and so we also performed the real-world analysis of our experience and confirmed that deployment was cost-saving during the first two UK waves (Figure 3).

Sustainable solutions for PPE will be an important consideration for healthcare services in the medium and long term, especially in resource-limited settings (10). SARS-CoV-2 is predicted to become an endemic, seasonal pathogen (6) requiring long-term PPE strategies, as it is likely that HCWs will be at high risk of exposure to SARS-CoV-2 during seasonal peaks of disease for the foreseeable future. This would also provide significant additional protection from other respiratory pathogens, including inevitable future influenza pandemics. Based on our evaluation, widespread respirator use can benefit staff, patients, the economy and the environment, and also associate with low staff absence rates compared to comparator institutions, with initial inpatient mortality data suggesting our hospital had relatively good outcomes. Healthcare settings should consider investing in PAPR systems such as the PeRSo for HCW who are at high risk of exposure to SARS-CoV-2, such as those working in intensive care, emergency medicine and acute specialties at risk of aerosol spread infections.

In terms of limitations, our survey had a proportionately low response rate (18% for standard PPE and 22% for PeRSo), which is unsurprising given the pilot deployment occurred during an intense period of clinical activity in the hospital. Many staff were re-deployed and circumstances changed daily. It is possible that respondents were not generally representative if they were the most motivated users (either positively or negatively), but survey responses were not suggestive of extreme opinions in either direction, and the real-word evaluation in wave 2 supported the conclusions. Our survey sample was predominantly female, and a large proportion was nursing staff. This gender balance is relatively representative of patient-facing staff, and it is unlikely that there are significant male-specific issues that were overlooked. However, one benefit is that beard-wearers can use a PAPR, while FFP3 masks require shaving (25), which may not have been captured. The number of survey respondents available for a paired analysis was low for the airborne PPE, due to the areas that the respirators were deployed until regulatory approval was given, which caused the statistical analysis to be underpowered for these differences. Invitations for the survey regarding the PeRSo were issued after 72 h of usage. As most negative comments were regarding the added difficulty for donning and doffing, this may have become a more important issue over time with repeated use and affected the overall impression of the PeRSo. We were unable to evaluate the potential for fomite transfer/contamination during the donning and doffing process, although this was not reported to cause significant difficulty in the user feedback. Longer-term issues, including skin changes, dry eyes, or other hearing impairments, would not be detected during our rapid feedback process, and longer-term evaluation will be necessary.

The simulation exercise hinges on several assumptions, which are listed in the Supplementary Appendix. One of the most important limitations of the economic analysis is that it can only account for the financial costs of adoption of PeRSo vs. PPE, but does not consider many aspects related to productivity impact and non-financial implications. The only productivity aspect that has been included in the analysis is the time “‘lost” in putting on and taking off PeRSos. The adoption of PeRSo could impact productivity in other ways. For example, the productivity of staff could be affected in a positive manner (e.g., due to reduced staff absence or increase in perceived safety) or in a negative manner (e.g., weariness from the background noise generated from the PeRSo). Furthermore, the analysis considers a time horizon of 360 days, which is likely to provide a “lower bound” for the longer PeRSo net cost saving, to the extent that PeRSo equipment would need no replacement before a few years. All in all, these limitations suggest that the analysis provides conservative estimates for PeRSo benefits.

In the staff absence analysis, these data are confounded by the fact that the absences include individuals not attending work as a result of isolation due to a family or social contact. Therefore, not all absences are due to staff infection. This confounder would be likely to reduce any impact of respirator use, not increase it, and the rapid fall in staff absences we observed is consistent with reduced staff infection rates at work. Ideally, ward-by-ward staff absence data would be collected and analyzed against confirmed infectious cases alongside different PPE provisions, but this requires prospective analyses that were simply not possible during the pandemic situation. Therefore, we can only report an “association” with low staff absence, not prove that respirator use directly causes reduced absence. However, these data refute initial concerns that widespread respirator use may lead to superspreader events and high levels of staff absence.

## Conclusions

The PeRSo was successfully deployed within 6 weeks during the acute first phase of the UK COVID-19 pandemic and used very widely in the second phase. This alternative PPE was preferred by HCW for its comfort, for the feeling of safety, and overall experience, helping to alleviate the high levels of stress and anxiety occurring during the COVID pandemic (26). PeRSos were the PPE preferred by patients as they allow the patient to see their carer's faces. Economic analysis indicates that widespread respirator use is a highly cost-effective and sustainable PPE solution, with greatly reduced environmental impact relative to disposable masks. Analysis during the larger second wave showed high staff uptake and low staff absence. Given the high likelihood of SARS-CoV-2 becoming an endemic, seasonal virus (6), healthcare institutions should consider investing in PAPR systems for long-term protection of HCW at risk of exposure to SARS-CoV-2, resolving issues around communication, staff anxiety, and supply chain issues of traditional PPE.

## Data Availability Statement

The raw data supporting the conclusions of this article will be made available by the authors, without undue reservation.

## Ethics Statement

Ethical review and approval was not required for the study on human participants in accordance with the local legislation and institutional requirements. Written informed consent for participation was not required for this study in accordance with the national legislation and the institutional requirements.

## Author Contributions

AM, JP, PE, and SF designed the service evaluation study. AM and MD collected the data for the service evaluation. AM undertook data analysis of the service evaluation. EM and CG undertook the economic evaluation. AM drafted the manuscript. All authors were part of the PeRSo implementation team, reviewed the manuscript, and approved submission.

## Funding

This study was supported by staff employed by the NIHR Southampton Clinical Research Facility (AM and SF). SF was an NIHR Senior Investigator. PE was supported by MR/P023754/1. The funders had no input into the study design or analysis.

## Conflict of Interest

The authors declare that the research was conducted in the absence of any commercial or financial relationships that could be construed as a potential conflict of interest.

## Publisher's Note

All claims expressed in this article are solely those of the authors and do not necessarily represent those of their affiliated organizations, or those of the publisher, the editors and the reviewers. Any product that may be evaluated in this article, or claim that may be made by its manufacturer, is not guaranteed or endorsed by the publisher.
