# Supplementary material for: Powered Respirators Are Effective, Sustainable, and Cost-Effective Personal Protective Equipment for SARS-CoV-2
Source: Front Med Technol. 2021 Oct 14;3:729658. doi: 10.3389/fmedt.2021.729658 (PMC8757687; doi:10.3389/fmedt.2021.729658)

Supplementary appendix

Contents

Economic evaluation parameters 2
PeRSo net-position simulations 2
Parameters of the cost calculation of PeRSo 2
Supplementary figure A 4

Economic evaluation parameters

PeRSo net-position simulations

The net position simulations are based on a number of parameters -- some fixed and some random -- following predefined distributions and have been apply to an intensive treatment unit (ITU) setting that resembles the set-up at the University Hospital Southampton (UHS). All random variables follow uniform distributions denoting very high levels of uncertainty.

Staff is fixed at 180 members, and is composed by 3% consultants, 13% junior doctors, 80% nurses and 3% physiotherapists. The number of beds available in the unit follows a discrete distribution between 5 and 40 patients (in steps of 5). Finally, the time horizon of the simulation exercise is between 10 and 360 days (in discrete steps of 10 days).

Parameters of the cost calculation of FFP3 facemasks

- Staff are assumed to work 5 of the 7 days per week and hence; in other words, on any given day about 70% of the 180 staff are present in the ITU
- Every member of staff present in the ITU is assumed to use 2 facemasks per day for their day-to-day activities in the Unit (i.e., for activities not in contact with patients).
- Our real-world hospital data analysis demonstrates that with sessional use, 14 FFP3 masks are used per day per patient. For the modelling, the number of times each patient is visited by a staff member follows a uniform distribution in the interval 8 to 16 times per day. A new facemask is required for every visit and is discarded after the visit.
- Following medical guidelines, for each visit a plastic visor is also worn and is changed after every visit. Visors are discarded every five days of use.
- Pricing for masks and visors follows current purchasing costs for UHS. FFP3 facemask prices are assumed to follow a uniform distribution in the interval £2.75 to £3.25, and visors similarly in the interval £0.8 to £1.2.
- Disposal costs for FFP3 masks are calculated from the current actual cost of waste disposal at UHS. Currently, UHS disposes equipment at £400 to £600 per tonne. Given that an FFP3 mask weighs 15gr (i.e. 66,667 masks per tonne) the disposal cost per mask is assumed to follow a uniform distribution in the interval £0.006 and £0.009. Similarly, visors weigh 6gr with disposal costs following a uniform distribution in the interval £0.002 and £0.004.

Parameters of the cost calculation of surgical facemasks

The scenarios for surgical facemasks follow the exact same processes as with FFP3 facemasks, with differences in their costs and weight. Surgical facemasks' unit costs is assumed to follow a uniform distribution in the interval £0.02 and £0.04, while their disposal costs (given their weight of 4gr) also follows a uniform distribution in the interval £0.0016 and £0.0024.

Parameters of the cost calculation of PeRSo

- Two possible prices are used for PeRSo given the current and future pricing strategies, i.e. £250 and £325.
- The running costs of PeRSO consist of: filter, pre-filter, and hood replacement (which takes place every six-months). Each respective cost follows a uniform distribution with the cost of a filter between £7 and £10, the cost of a pre-filter between £0.8 and £1.2, the cost of a hood between £1 and £2.
- Disposal costs also follow uniform distributions with their ranges obtained given their weight. The assumption is that the PeRSo hood, filter and pre-filter each weigh as much as

20 FFP3 with disposal cost in the interval £0.12 and £0.18 each. The PeRSo hood is assumed to resemble the stand-alone visor and the disposal cost is between £0.002 and £0.004 (also following uniform distribution).

- Daily use of PeRSo requires each staff member to take between 20 and 30 minutes per day (assumed random from a uniform distribution) to put it on and take it off. These values are translated into productivity loss through a weighted average salary of the ITU staff (i.e., Consultant at £95,000, Junior doctors at £35,000, Nurses at £30,000, and Physiotherapists at £30,000). Furthermore, staff work 46 weeks per year and 37.5 hours per week.
- Finally, a member of staff is assumed to be hired with annual salary of £20,000 to attend to the PeRSO devices, clean them and prepare for daily use such as charging.

Total cost of FFP3 facemasks scenarios

- Total cost of FFP3 facemasks used is taken by adding up the per day personal FFP3 masks used by staff in the ITU, all FFP3 facemasks and visors used during patient visits, and the disposal costs of all such FFP3 facemasks and visors. This total daily cost is then multiplied by the horizon of each scenario in order to obtain the cumulative costs.

Total cost of surgical facemasks scenarios

- Total cost of surgical facemasks follows the exact same fomula as above but replaces FFP3 with surgical facemasks and their costs.

Total cost of mixture FFP3 and surgical facemasks scenarios

- Total cost of mixture masks scenario is calculated to allow for mixture of FFP3 and surgical facemasks usage. The proportion of FFP3 facemasks used follows a uniform distribution varying between 60% to 90% (i.e., respectively surgical facemasks are between 10% to 40%) with costs proportionally applied.

Total cost of PeRSo scenarios

- Total cost of PeRSo is calculated by summing the total fixed-cost from the purchase of 180 devices (i.e., full purchase cost take place on the first day) plus the daily cost of use and disposal of each PeRSo and assuming replacement of parts every 6 months plus daily cost of the PeRSo care-taker plus the productivity losses from time lost in using the PeRSo. With the exception of the initial fixed cost, all daily components are multiplied by the horizon days in each scenario.

Net-positions

- Simulations examine cost savings from PeRSo use in a head-to-head comparison of a) FFP3 against PeRSo and b) Mixture FFP3/surgical facemasks with PeRSo. Simulations are performed over a time horizon of 10 to 360 days and a range of 5 to 40 patients in the ITU. Separate calculations are presented for two possible prices of PeRSo, with 10,000 simulations for each price level.

Supplementary figure A

Patient feedback questionnaire graphic

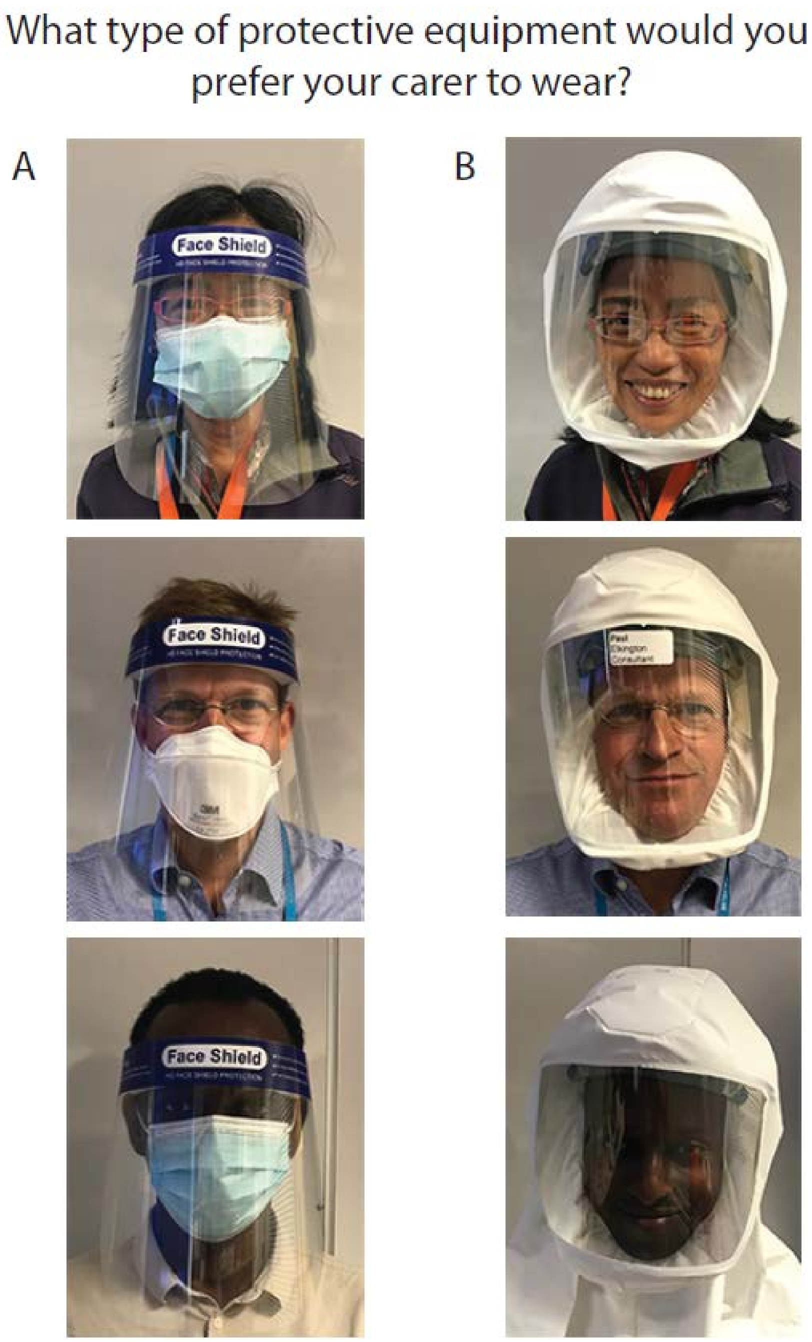

Supplement: Supplementary file 1 [file Data_Sheet_1.PDF]
